# Supplementary material for: Are carbon emissions trading and green financial instruments synergistic? -Comprehensive quantitative research based on content analysis
Source: PLoS One. 2024 Mar 7;19(3):e0298601. doi: 10.1371/journal.pone.0298601 (PMC10919601; doi:10.1371/journal.pone.0298601)
Supplement: S1 Data — (ZIP) [file pone.0298601.s001.zip › Data/地级市Z_F工作报告.zip!/╝¬┴╓/╦─╞╜╩╨/└┤╫╘╣1⁄2╓┌║┼í╛┬φ┐╦╩2╛▌═°í┐_2006.docx]

各位代表：

　　现在，我代表四平市人民政府，向大会报告政府工作，请予协商讨论，并提出意见。

　　一、2005年工作回顾

　　过去的一年，在市委的正确领导下，坚持用科学发展观和振兴老工业基地统领各项工作，全力推进具有全局性和战略性的重点任务，真抓实干，克难攻坚，全市经济社会呈现快速直辖市推进、持续健康增长的强劲势头，全面完成了“十五”时期确定的各项目标任务。全市生产总值预计完成330亿元，同比增长18.9%；一、二、三产增加值分别完成125.6亿元、98.23亿元、106.7亿元，同比分别增长12.4%、22.6%、23.9%；全社会固定资产投资预计完成86.5亿元，同比增长57.2%；社会消费品零售总额实现100亿元，同比增长15%；一般预算全口径财政收入完成19.15亿元，同比增长15.7%，其中市区财政收入完成9.74亿元，同比增长20.4%，是11年以来增长幅度最高的一年。

　　——项目建设成果显著。全市在建项目360个，投资总额255亿元，完成投资68亿元。投资亿元以上项目59个，同比增长96.7%，竣工8项，完成投资14.5亿元。实施技改项目65项，完成技改投资30亿元。中粮黄龙工业园、福耀660吨级浮法玻璃、红钢100万吨中宽带钢、通钢20万吨冷轧带钢、昊华6万吨离子膜烧碱、天成30万吨玉米深加工、庆达可录光盘等重点项目，将成为拉动我市经济快速发展的增长点。15个第一、第二批国家振兴东北老工业基地项目总投资30.96亿元。国债投资项目27个，争取国债资金2.57亿元。争取开行贷款3亿元。这是我市实施项目带动战略以来项目建设形势最好的一年。

　　——国有工业企业改制任务基本完成。全市共完成153户国有企业改制任务。列入省计划的89户国有工业企业基本完成改制，商业等其它国有企业完成改制64户。粮食系统全面完成改制任务。共化解金融债务23亿元，解决职工债务3亿元，偿还承债社保本金2亿元。市工商银行剥离不良贷款28.9亿元，市城市信用联社一次性卸掉历史包袱3.3亿元。土地转让收入用于改制成本2.1亿元。全市筹集并轨资金8.2亿元，完成并轨人数10.1万人，占国有企业下岗失业人员总数的60%。市热力总公司改制取得圆满成功，‘采取公开竞价方式出售，市财政收回资金4368万元。改制后服务质量显著提高，供热情况也好于上年，热费在全省普遍上调的情况下，新的热力公司保持不变，使人民群众的利益得到了保障。联合化、收割机、油脂化、钢丝绳等一批困难企业在改革中重获新生。通过改制，工业经济的质量和效益明显提高。预计全市规模以上工业企业完成增加值68亿元，同比增长26.9%。工业经济在2004年扭转连续10年亏损的情况下，去年又实现利润3亿多元。全市民营经济增加值和上缴税金分别达到90亿元和6.4亿元，同比分别增长26%和20%。两大主导产业规模以上企业已发展到138户，占规模以上企业总数的49.8%，完成工业总产值占全市工业总产值的54.4%。连续两年的盈利和良好的发展势头，标志着我市工业经济已经走出低谷，开始进入良性循环的轨道。

　　——农村经济全面发展。全市粮食产量达到118.1亿斤，继2004年之后又创历史新高。猪、牛、羊、禽饲养量分别达到805万头、248万头、185万只和1.48亿只，同比分别增长13.8%、7%、7.9%和7.4%。牧业总产值112.6亿元，同比增长5.5%。禽流感防控工作取得阶段性成果，实现了“四无”目标。农产品加工业实现产值65亿元，同比增长14.4%，占全市规模以上工业总产值的38%。全市农业标准化示范区达到85个，建设标准化生产基地400万亩。二龙山等11座大中型水库除险加固进展顺利，总蓄水量达到10.5亿立方米，二龙山水库已经达到防汛水限，是11年来蓄水量最多的一年，将对全市工农业生产和项目建设起到巨大的保障作用。全年转移农村劳动力40万人（次）以上，劳务经济收入超过11亿元。农村新型合作经济组织增至1020个，带动农户12.7万户，人口44.5万人，均占农户和农业人口的四分之一。认真落实促进农民增收的各项政策措施，免征农业税配套改革和国有农场税费改革基本完成、国家给予我市农业税转移支付、粮食直补、产粮大县奖励和三项技术补贴等资金7.1亿元。

　　——招商引资成效明显。全市共引进招商引资项目886个，续建项目123个，实际到位资金86.4亿元，同比增长65.8%。省外项目到位资金30.6亿元，是省下达计划的2.2倍。工业项目和农业产业化项目所占比重达到75%以上，同比增加15个百分点。合同利用外资8643万美元，实际利用外资1766万美元，同比增长59.4%。各开发区和工业集中区共引进开发项目187个，到位资金32.6亿元，占全市总数的37.7%。对外交流不断扩大，与南昌市及俄罗斯白山市、韩国机张郡缔结为友好合作城市。组织参加了四平——香港项目推介会、苏沪浙经贸交流会、东博会、哈洽会、西洽会等招商引资活动，共签订合同项目85个，金额达87.3亿元。引进项目履约资金到位率明显提高，四平——香港项目推介会签约18个项目，已有11个项目开工建设。

　　——基础设施和城市环境建设取得新突破。城区共完成基础设施建设投资15.38亿元，投资额度、项目数量和建设速度均创历史新高。新建城市道路18条。影响平东工业区发展的平东大路、平东南路建成通车。英雄大街西段工程高标准高质量的完成，使英雄大街向西延伸1.17公里，与环城路相连通，为打造精品街路奠定了基础。北环路和102国道、四叶一级路改造工程进展顺利，四平的交通枢纽和辐射功能将进一步增强。北迎宾街公铁立交桥建成投入使用，为红嘴集团和红嘴高新技术开发区的发展创造了良好环境。民营企业家捐资赞助，修建巷路、人行步道38条，进一步解决了群众出行不便的困难。火车站、站前广场和周边改造工程基本完成，站前广场已成为我市一道新景观，一个美丽现代化的“窗口”展现在世人面前。宏泰公园、热电广场周边绿地主体工程圆满竣工，铁东区居民多年期盼的铁东公园建成，结束了铁东区没有文化娱乐广场的历史。继南河改造后，北河歇造工程全面启动，完成了10.9公里的北河护岸挡土墙和6公里的污水截流干管工程。两岸各30米绿化带和6米机动车道建设用地的拆迁工作已经完成，共拆迁房屋面积23775平方米。绿化覆盖率明显提高，年内新增绿地56.23万平方米。“两河、四场、八街”亮化工程取得明显效果，四平的夜间景观也初步展现出现代化的魅力。广电大厦、四平战役纪念馆、传染病医院等工程完成计划进度。以“五项整治”、“三项建设”为主要内容的城区环境综合整治取得明显成效，国家卫生城市成果不断巩固和发展，全国北方国家卫生城市现场经验交流会在四平成功召开。全年完成交通建设投资13.2亿元，竣工公路677.6公里，村村通油路率达到62.8%。二龙山水库污染综合治理取得实质性进展，水质得到较大改善。

　　——群众生活水平不断提高。预计全市城镇居民人均可支配收入8550元，同比增长10%；农民人均纯收入达到3400元，同比增长11%。全年创造就业岗位6.1万个，新增就业5.2万人，下岗失业人员实现再就业3.4万人。社会保障工作实现重大突破，社会保障能力显著提高，全市社保自有资金达到2.6亿元，创造了我市社保自有资金积累历史最高峰。全市共拨付养老金、失业金5.85亿元，及时足额发放率达100%，退休人员社会化管理率达到96%。在全省率先开展了农村医疗保障试点。城市享受低保人数达到10.2万人，发放最低生活保障金8220万元，社会化发放率达到100%。

　　——各项社会事业协调发展。国家和省批准重大科技项目30项，启动实施市级科技攻关项目100项。申请国内专利75项。25户企业被确定为省级科技企业。“以县为主”的农村义务教育管理体制进一步完善，控制学生流失工作取得新成果，启动实施职业教育“百强校”建设和千名骨干教师培训工程，“经历教育”在全市中小学全面展开。“评聘结合”试点工作圆满完成，国家在我市召开了现场会。与北京市签订科技教育合作协议。成功举办党政军民迎国庆大型文艺演出，积极开展“英雄城古今”大型百年展活动。医疗体制改革不断推进，新型农村合作医疗试点工作成效明显，参合率达到76.7%。举办了第四届社区体育大赛，我市运动员在第十届全国运动会上获得3枚金牌、2枚银牌、2枚铜牌的好成绩。全国经济普查工作获得国家表彰，1%人口抽样调查工作进展顺利。实现土地收益2.6亿元，查处各类土地违法案件119件，保证了重点项目建设用地需要。广播电视基础设施得到根本改善，节目质量不一断提高。人口与计划生育工作取得新进展，计生线、党政线考核均居全省前列。双拥模范城成果进一步巩固，吉林省保交护路四平民兵训练基地建成投入使用。档案工作取得新突破，市档案馆通过全省首批首家3A级验收。民族宗教、侨务、旅游、人防、地震、气象、老龄、地方志和残疾人等各项工作取得新的成绩。

　　——民主法制和政府自身建设进一步加强。自觉接受人大的法律监督、工作监督和政协的民主监督。全年办理人大代表建议116件，政协委员提案159件，办复率达100%。围绕创建“平安四平”的目标，深入开展严打整治斗争，共破获各类刑事案件4480起，破案率提高24.8%，社会治安形势稳定。“四五”普法任务顺利完成。全市安全生产形势良好，未发生重特大安全生产事故。认真解决和处理信访突出问题及群体性事件，国家和省交办信访案件办结率达到100%。深入贯彻落实《行政许可法》，实行行政审批时限制，审批时间由原来平均22个工作日减少到5个工作日。深入开展政务公开，率先在全省建成市、县两级政府政务大厅。通过开展文明执法“双标兵”争创活动以及查办一批违法违纪案件，加强了经济发展的软环境建设。

　　各位代表，这些成绩的取得，是市委统揽全局、科学决策的结果，是各地各部门全面落实科学发展观、深入实施项目带动战略的结果，是全市各族人民同心同德、共同努力的结果。在此，我代表市人民政府，向奋战在全市各条战线的广大劳动者，支持政府工作的市人大、市政协，各民主党派、工商联、群众团体，向关心帮助四平市建设的驻平部队、武警官兵、中省直单位及海内外朋友表示衷心的感谢！

　　回顾一年来的工作，我们也清醒地看到，我市经济和社会各项事业发展中还存在一些比较突出的问题，主要是经济总量不大、结构不优、效益不高、市场化水平低、自主创新能力不强；促进农民增收的长效机制还没有形成，农业综合生产能力还需要进一步提高；财政支撑保障能力不强，就业再就业、社会保障压力较大，困难群众生产生活问题需要下大力气解决，维护社会稳定和化解各种矛盾的任务繁重；部分干部思想、观念、作风与新形势、新任务的要求还不完全适应等等，这些问题需要在发展过程中逐步加以解决。

　　二、2006年主要任务

　　2006年是实施“十一五”规划的开局之年，也是振兴四平老工业基地进入关键的一年。我们要充分利用“十五”以来形成的较好发展基础和国家振兴东北战略，积极面对转变经济增长方式和加快发展的压力，增强责任感和危机感，突出发展重点，把握发展主动权，为顺利实施“十一五”规划打下良好基础。今年工作的总体思路是：以邓小平理论和“三个代表”重要思想为指导，认真贯彻党的十六届五中全会、省委八届八次全会和市委四届五次全会精神，用科学发展观统领经济社会发展全局，继续全面实施项目带动战略，加快改革开放步伐，全力推进招商引资，着力转变经济增长方式，切实优化经济结构，提高发展速度和质量，大力推进工业新型化、农业产业化和城乡一体化。坚持以人为本，充分关注民生，构建和谐四平，加快新型工业城市和社会主义新农村建设．全面完成“十一五”开局之年的各项目标任务。

　　主要预期目标是：全市完成生产总值385亿元，增长16%以上；一般预算全口径财政收入22亿元，增长15%以上；全社会固定资产投资完成117亿元，增长35%；城镇居民人均可支配收入实现9660元，农民人均纯收入3700元，分别增长13%和9%以上；城镇登记失业率控制在4.7%以内；居民消费价格指数为102%；人口自然增长率控制在4‰以内。

　　实现上述目标，要重点抓好以下工作：

　　（一）深入实施项目带动战略，确保固定资产投资高速增长。

　　抓紧落实“十一五”规划重大项目，突出工业项目和企业技改项目，发展“十大产业”，培育“五个基地”。计划安排500万元以上项目261项，其中续建项目113个，新开工项目87个，前期工作项目61个。突出抓好四平热电厂二期、双辽电厂二期、大唐风力发电、红钢中宽带钢、通钢四平钢绞线和精密冷轧带钢等重点项目。高质量完成前两批老工业基地项目和国债基础设施项目，做好第三批老工业基地项目和新建续建国债项目的争取工作。积极支持哈大客运专线建设，抓好省建交通、能源等重大项目。积极培植符合开行贷款条件项目，进一步扩大开行贷款额度，拓宽贷款投入领域。扩大招商引资和民间投资，实现重大项目投资主体多元化。顺应国家宏观调控措施，用足用好各项政策，围绕国家扶持重点领域和我市主导产业及优势产业，精心策划、论证、包装一批带动作用强、辐射范围大、发展后劲足的大项目，严格落实项目包保和管理责任制，提高项目质量和效益。

　　（二）切实转变经济增长方式，加快新型工业化进程。

　　全市规模以上工业企业总产值和增加值要确保增长21%和16%以上。强化工业经济运行调控、调度和预警预测，加强煤、电、油、运、气等生产要素的衔接和组织，对年产值5000万元以上重点企业加强支持和指导。突出抓好企业技术改造，技改投资增长35%以上。推进企业技术进步，加快企业技术中心建设。积极应用先进适用技术提高工业经济整体技术水平，加快企业新产品开发，新产品产值增长20%以上。大力实施质量兴市战略，鼓励和支持企业争创名牌。加快产业结构调整和优化升级，加大市场开拓力度，提升产业竞争力和自主创新能力。推进企业节能降耗，确保完成省里提出节能降耗30%的任务。推行清洁生产，实现循环利用，努力发展资源节约型和环境友好型工业。切实加强企业管理，坚决扭转一些企业效益低、质量差、能耗高的状况。进一步发展壮大民营经济。培植一批具有聚集优势、核心竞争力强的重点民营企业，使民营经济成为推动老工业基地振兴的生力军。大力推进中小企业信用担保体系和服务体系建设，完善市县两级担保机构和中小企业服务中心，建立中小企业发展墓金。积极推进创建投资公司试点，拓宽民营企业融资渠道，为民营经济提供及时有效的服务。全市民营经济营业收入和增加值要增长25%以上。

　　（三）积极推进社会主义新农村建设，提高农村经济发展整体水平。

　　按照“生产发展、生活宽裕、乡风文明、村容整洁、管理民主”的要求，努力实现社会主义新农村建设良好开局。以建设现代农业为目标，加快农业和农村经济结构调整，大力发展非农产业，推动农村工业向园区集中，农业向优势品种集中，土地向规模经营集中。大力推进农业产业化经营和标准化生产，稳定提高粮食综合生产能力，实施“5841”优质粮食工程，加强产粮大县和优质粮食基地建设，积极争取上级农林水等项目和资金支持。动员社会力量参与，加快标准化高产农田建设，促进生态环境改善。优化配置牧业要素，打造一批专业化、规模化和标准化牧业小区，认真搞好动物防病防疫，加快推进牧业经济强市进程。以省里建设“长一吉一平”粮食和畜产品加工产业密集带为契机，全力扶持农产品加工企业改建扩建，培育农产品加工龙头企业。适应农业现代化和农村工业化的要求，加快综合性、区域性农产品批发市场建设。大力发展各类协会和专业合作经济组织，提高农业市场化水平。加快农村富余劳动力输出和转移，搞好专业性和适应性职业技能培训，提高农民劳务输出规模和质量，力争实现50%的农村劳动力离粮、离土、离乡。以建设文明生态村为重点，分期分批规划和建设新村屯，努力形成分工明确、功能互补、配套完善、环境优美、协调发展的新格局。建设农村公路600公里以上，村村通油路率达到77.7%。加快发展小城镇，引导周边村屯向乡镇集中，不断扩大乡镇规模，提高城镇承载能力。健全各项民主制度，保障村民对新农村建设的知情权、参与权和监督权，全面完成建设社会主义新农村试点任务。

　　（四）全面推进改革开放，不断增强发展的动力和活力。

　　巩固国有工业企业改革成果，按照“四到位”标准组织开展回头看，认真解决改革遗留问题。积极利用开发银行贷款，满足企业并轨资金需求。落实各项改制政策扶持措施，把改制企业培育成充满活力的市场主体。搞好厂办集体企业改制一试点。推进商务、建设、房产、公用、交通等系统的国有企业改革，基本完成非工国有企业的改制任务。全面推进事业单位改革。完善扩大县（市）经济社会管理权限的各项配套措施，继续深化乡镇机构、农村义务教育和县乡财政管理体制等综合配套改革。深化金融体制改革。大力发展信用中介机构，提高企业诚信度，化解不良资产，维护金融债权。金融部门要进一步扩大信贷规模，增加有效信贷投入，促进全市工农业生产发展和项目建设。继续加大招商引资力度。坚持“走出去、请进来”，创新招商方式，提高组织层次，继续落实招商引资工作责任制，提高招商引资的质量和水平。积极组织参加第二届东博会以及珠三角、长三角、环渤海湾、欧盟地区等经贸招商活动。全年力争完成招商引资任务100亿元。提高开发区和工业集中区建设水平，进一步搞好园区规划和布局，加快基础设施建设，提高配套服务质量，完善灵活高效、安商富商的管理运行机制和政策，增强产业集聚和资本吸纳能力。做强做优四平经济开发区、红嘴高新技术开发区，加快铁东经济开发区、吉林四平循环经济示范区、机械工业园区和食品工业园区建设速度。打牢各工业集中区基础工作，力争进入省级开发区行列。积极开展对外贸易，大力实施市场多元化和以质取胜战略，鼓励企业参与国际市场竞争，优化进出口商品结构，提高对外贸易的质量和效益，增强出口创汇对经济发展的拉动作用。

　　（五）实施城市棚户区改造工程，全面加强城市建设与管理。

　　完成近期城市建设规划编制工作，做好新一轮土地利用总体规划修编。今年城市建设要突出抓好三件事：一是抓好城市棚户区改造工程。用三年时间全面完成棚户区改造任务，今年要完成拆迁改造50万平方米以上。三年后建成区内的人民群众彻底告别棚户区，五年内绝大多数棚户区的群众住进现代化的居民小区。二是抓好土路、巷路改造工程。硬面化改造土路、巷路10万延长米、52万平方米，铺装人行步道5400延长米、2万平方米，在民营企业捐资修建38条土路和巷路的基础上，年内基本实现建成区内土路的硬面化，让人民群众彻底告别土路。三是抓好亮化工程。在前年“两街”30个亮化试点和去年“两河、四场、八街”亮化工程的基础上，今年全面实施亮化工程，建成区内主干路、部分次干路和所有广场要全面亮化，使四平这座城市不但要绿起来，美起来，也要亮起来。同时要完成垃圾无害化处理厂建设工程，启动污水处理厂，筹建中水回用工程。改造城市供水管网，完善供热管网，更新临界期燃气管线。完成交通建设投资12.35亿元，重点建设四平环城路北段、四叶一级路、烟厂路四梅铁路公铁立交桥，改造102线城区南北出口。继续巩固和提高国家卫生城市成果。进一步理顺城市管理体制，探索综合执法办法，提高管理效能。积极启动国家园林城市创建工作。在城市环境绿化、净化、美化、亮化的基础上，努力提高园林艺术水平，完善居民小区、学校、庭院绿化，增加绿量，提高水平。北河滨河公园开工建设两岸各30米绿化带和6米机动车道，竣工后人们可以驱车游览两岸风光。抓好铁东公园、宏泰公园、热电广场周边绿地、西郊广场及市政道路绿化，新增绿地32万平方米。加大环境保护执法力度，继续推进二龙山水库污染综合治理。

　　（六）深入实施科教兴市战略和人才强市战略，推进社会事业全面进步。

　　今年要组织申报省级以上科技大项目35项以上，继续抓好高斯达金属纳米粉制取设备技术及产业化等10个高新技术产业化项目，加快高新技术企业创业服务中心和国家级生产力促进示范中心建设步伐。以促进发展为根本出发点，紧紧抓住人才培养、吸引、使用三个环节，加强经营管理人才、专业技术人才、高技能人才和农村实用人才队伍建设。加强农村基础教育，巩固“普九”成果，积极发展职业教育、民办教育和高中阶段教育，深化中小学生“经历教育”，加强师资队伍建设，加大教育投入，推进教育信息化，积极发展现代远程教育。加快吉林师范大学应用工程学院建设，充分利用和优化高等教育资源。积极发展文化事业，加大文化市场的监督管理力度，大力扶持公益性文化事业，积极开发文化产业。继续加强公共卫生体系建设，完善公共卫生应急体系，提高应对突发公共卫生事件能力，抓好卫生执法监督、疾病预防控制、城镇医疗体制改革和农村卫生工作，进一步完善新型农村合作医疗试点。切实加强食品药品安全监督管理工作。加快广电大厦建设进度，深入实施精品战略，促进广播电视事业健康发展。努力构建全民健身服务体系，增强竞技体育综合实力，积极筹备四平市林育场馆建设，力争启动体育场建设。加快建立人口和计划生育工作新机制，落实好农村部分计划生育家庭奖励扶助制度和城镇独生子女父母奖励政策，深入开展计划生育“三结合、五关爱”活动。大力推进社会主义精神文明建设。深入开展“双拥”活动，巩固双拥模范城成果。继续做好民族宗教、外事、侨务、旅游、人防、地震、老龄、地方志、知识产权、档案管理、残疾人救助等工作。

　　（七）高度关注民生，建设和谐四平。

　　千方百计扩大就业。进一步落实再就业扶持政策，开发社区和小城镇就业岗位，培育、引导、扶持下岗失业人员自谋职业和自主创业，解决好“4050”人员再就业问题。加快“金保工程”建设，完善城乡就业一体化的人力资源市场体系，推动城乡劳动力合理流动。加快完善新型社会保障体系。进一步做好城市低保，完善城镇职工基本养老和失业、医疗、工伤、生一育保险等制度，探索建立农村社会保障制度，扩大社会保险覆盖面。加强社会救济和救助工作，及时解决灾民和困难群众的生活问题。加强和改进信访工作。进一步贯彻《信访条例》，建立和完善信访工作长效机制，依法规范信访秩序，提高基层处理信访问题能力，完善大信访格局，真正解决企业改制、土地纠纷、拆迁安置、涉法涉诉等涉及群众切身利益的突出问题。切实做好安全生产工作。坚持安全第一，预防为主，加强依法监管和专项整治，提高对突发安全生产事件的应急处理能力，实现全市无重大安全生产事故目标。高度重视消防工作，加强重点部位和重点行业检查，坚决杜绝重特大恶性事故发生。进一步做好防灾减灾工作。办好市长公开电话，及时解决群众反映的问题。整顿和规范市场秩序，严厉打击各类违法行为。深入推进社会治安防控体系建设，依法严厉打击各种刑事犯罪活动，维护全市政治安定和社会稳定，创建平安四平。

　　（八）提高行政能力，建设服务型政府。

　　加快政府职能转变。履行好经济调节、市场监管、社会管理和公共服务职能，深化政务公开，加快发展电子政务，为人民群众提供公开、透明、高效的公共服务。加快行政管理体制和行政审批制度改革，高标准办好政务大厅，深入实施行政审批时限制，推动政府工作增速提效。大力开展增收节支，夯实收入基础，建立健全公共财政体系。

　　切实改进工作作风。继续精简会议、减少文件，集中精力抓大事、抓实事、抓落实。各级干部要重实际、重实干、重实效，积极主动地为基层、企业和群众解决具体问题。强化政府机关问责制，进一步解决不作为和乱作为的问题。树立正确政绩观，不搞形式，不图虚名，把主要精力投入到深入实际、破解难题、推动发展上来。

　　全面推进依法行政。深入贯彻《行政许可法》启动“五五”普法教育，推进法治政府建设。加强行政执法队伍建设，文明执法，热情服务。主动接受人大的依法监督、政协的民主监督，认真接受社会公众和新闻舆论的监督。积极办理人大议案、建议和政协提案。加强同各民主党派、群众团体、无党派人士的联系。充分听取社会各界的意见和建议，推进决策的科学化、民主化和制度化。

　　加强廉政建设和反腐败工作。完善惩治和预防腐败体系，强化行政监察和审计监督，督促落实省里下放的各项权力，重点对招商引资、行政执法、损害群众利益等突出问题进行监督检查，确保政令畅通。落实党风廉政建设责任制和领导干部经济责任审计制度，坚决查处违纪违法案件，严惩腐败分子。进一步加大软环境建设工作力度，为经济发展创造良好环境。全体公务员特别是领导干部要模范遵守党纪政纪，廉洁从政，树立和维护政府的良好形象。

　　各位代表！今年是“十一五”规划的第一年，“十一五”规划的宏伟蓝图催人奋进，新的事业激励我们奋力开拓。让我们在市委的正确领导下，凝聚全市各族人民的智慧和力量，解放思想，励精图治，真抓实干，为加快四平老工业基地振兴、为“十一五”规划目标的全面实现而努力奋斗！
